# Drugs available through the Farmácia Dose Certa program and Beers criteria: a further analysis

**DOI:** 10.1590/S1516-31802012000400012

**Published:** 2012-09-04

**Authors:** Márcio Galvão Oliveira, Welma Wildes Amorim, Luiz Carlos Passos

**Affiliations:** I BPharm, MSc. Assistant Professor of Clinical Pharmacy, Instituto Multidisciplinar em Saúde, Universidade Federal da Bahia (UFBA), Vitória da Conquista, Bahia, Brazil.; II MD. Geriatrics Specialist, Professor of Internal Medicine, Department of Natural Sciences, Universidade Estadual do Sudoeste da Bahia (UESB), Vitória da Conquista, Bahia, Brazil.; III MD, PhD. Adjunct Professor of Internal Medicine, Department of Internal Medicine, Faculdade de Medicina da Bahia (FAMEB), Universidade Federal da Bahia (UFBA), Salvador, Bahia, Brazil.

Dear Editor,

We read the recently published article by Lucchetti et al.^1^ and we have an important comment to make on this study. The objective was to assess the drugs in the Farmácia Dose Certa (“Right Dose Pharmacy”) program that were considered to be inappropriate for the elderly. The authors used the criteria developed by Beers and updated by Fick in 2003 to assess the appropriateness of drug use among elderly people.^2^ As described by the authors, the Beers-Fick criteria are divided as follows: (1) drugs or drug classes that should generally be avoided for people over 65 years of age, because they are ineffective or have a high risk of unnecessary adverse effects when a safer alternative is available; and (2) medications that should not be used for elderly patients with specific known conditions. However, only those medicines listed in the first group of Beers-Fick criteria were compared with the available drugs within the Dose Certa program. This underestimates the quantity of potentially inappropriate medications for older people available through this program. One example of this is that metoclopramide should be avoided among patients with Parkinson’s disease because of its antidopaminergic/cholinergic effects,^2^ but was not included in the analysis by Lucchetti et al.^1^

In our view, the main contribution of their study is to disseminate information about the medications that can potentially be considered inappropriate, and serve as a reference in daily clinical practice. In addition, this information may provide support for regional or national pharmacy and therapeutics committees in making choices of therapeutic alternatives that are more cost-effective and safer for the elderly population.
